# Integrating procurement, prescription, and resistance data to strengthen antimicrobial stewardship: insights from a public health institution in India

**DOI:** 10.3389/fmicb.2025.1673019

**Published:** 2025-10-31

**Authors:** Vinay Modgil, Sundeep Sahay, Arunima Mukherjee, Rashi Banta, Neha Joshi, Rashmi Surial, Subhash Thakur, Suvodeep Mazumdar, Sneha Roychowdhury, Neelam Taneja

**Affiliations:** ^1^Society for Health Information Systems Programmes (HISP India), New Delhi, India; ^2^Department of Informatics, Faculty of Medicine, University of Oslo, Oslo, Norway; ^3^Institute of Health and Society (HELSAM), University of Oslo, Oslo, Norway; ^4^School of Information, Journalism and Communication, University of Sheffield, Sheffield, United Kingdom; ^5^School of Computer Science, University of Sheffield, Sheffield, United Kingdom; ^6^Department of Medical Microbiology, Postgraduate Institute of Medical Education and Research, Chandigarh, India

**Keywords:** antimicrobial resistance, hospital, consumption, prescription, antimicrobial susceptibility testing, antimicrobial stewardship policy

## Abstract

**Introduction:**

Sustained and sub-optimal antimicrobial use drives antimicrobial resistance (AMR), a major health systems challenge in low- and middle-income countries (LMICs) such as India. This study examined the relationship between institutional antimicrobial procurement and outpatient prescribing patterns, and how these influence resistance trends identified through antibiotic susceptibility testing (AST) in a public community hospital.

**Methods:**

Data were collected from three sources: (i) procurement records (2018–2022), (ii) AST results from urine, pus, and stool samples (2023–2024), and (iii) outpatient prescriptions (2023–2024). Each dataset was analyzed individually and in an integrated framework to assess interrelationships between antimicrobial use and resistance.

**Results:**

Amoxicillin-clavulanate, ciprofloxacin, and doxycycline were among the most procured drugs, with Escherichia coli (urine) resistance rates of 53%, 87%, and 39%, respectively. The most frequently prescribed antimicrobials were Amoxicillin-Clavulanate (24%), Cefixime (15%), and Azithromycin (11%); over 50% were broad-spectrum agents and over 90% belonged to the WHO AWaRe “Access” category. Correlation analysis revealed a weak positive association between procurement and sensitivity, indicating that higher procurement did not necessarily increase resistance.

**Discussion:**

These findings demonstrate the feasibility of linking institutional datasets to identify inefficiencies in antimicrobial use and guide evidence-based stewardship interventions, including formulary revision, procurement alignment, and data-driven prescribing practices.

## 1 Introduction

The growing and rapid spread of antimicrobial resistance (AMR) presents an urgent threat to public health worldwide, with India being particularly at high risk. India faces an enormous challenge due to its large population, inadequate sanitary facilities, high rates of infectious diseases, and excessive use of antimicrobials (Ferrara et al., 2024). An estimated 297,000 deaths in India were attributed to AMR in 2019 (Lahariya et al., 2024), which is estimated to rise to 2 million deaths by 2050. This could be attributed to the high rate of bacterial infections, poor infrastructure, and uncontrolled antimicrobial use (Murray et al., 2022). Globally, deaths due to AMR are estimated to be 700,000 per year, which is projected to increase to 10 million by the year 2050 (WHO, 2019). The annual cumulative loss of economic output globally from AMR is estimated to amount to 20–35 trillion US dollars by 2050 (Dadgostar, 2019).

The antimicrobial ecosystem in India is characterized by unrestricted over-the-counter sales, large-scale manufacturing, the marketing of fixed-dose combinations (FDC), weak regulation, and ambiguities in responsibilities between national and state-level agencies that complicate antimicrobial availability, sales, and consumption (Fazaludeen Koya et al., 2022). Unregulated over-the-counter sales, the proliferation of fixed-dose combinations, weak regulatory oversight, and fragmented institutional responsibilities fuel the overuse and misuse of antimicrobials, thereby accelerating the development and spread of AMR. Antimicrobial sales data in India indicate that the use of newer classes of antimicrobials is disproportionately high compared to older, first-line antimicrobials recommended in essential drug lists and stewardship guidelines (Fazaludeen Koya et al., 2022). In India, human consumption of antimicrobials increased by 36% between 2,000 and 2,010, including 23% through retail sales. The Center for Disease Control (CDC), India, outlined an increased risk of AMR for the treatment of common diseases such as malaria, leprosy, and meningococcal infections. kala-azar, Tuberculosis, and HIV (Ganesh Kumar et al., 2013).

Increasing AMR has significant adverse implications on healthcare costs in addition to mortality and morbidity, particularly in disadvantaged populations (Allel et al., 2023; Chokshi et al., 2019). Existing research has focused largely on individual behavior (Bebell and Muiru, 2014), while studies at the level of community hospital antimicrobial procurement and prescription are limited (Fazaludeen Koya et al., 2022; Yin et al., 2018). The antimicrobials procured by a community hospital dictate what antimicrobials are prescribed and consumed, which impacts antimicrobial susceptibility testing (AST). Patients visiting public hospitals are expected to procure their prescribed (subsidized) medicines from government pharmacies and so, remain trapped within a cycle of what medicines are procured and prescribed by the hospitals. Developing an understanding of the relationship between antimicrobial procurement/prescription and AST patterns is critical for determining drivers of AMR, which can inform more effective antimicrobial stewardship management (Demoz et al., 2020).

Most existing studies in India and other LMICs have examined either antimicrobial consumption patterns or resistance surveillance in isolation, with limited attention to how these data streams intersect at the hospital level. By combining procurement, prescription, and AST data, our study seeks to capture these interconnections and provide a systems-level perspective on antimicrobial use. Such integration enables identification of mismatches between what is procured, what is prescribed, and what remains effective, thereby highlighting drivers of AMR that cannot be captured through siloed analyses. This rationale underlines the novelty and policy relevance of our study, offering insights that can directly inform the design of antimicrobial stewardship policies (Modgil et al., 2025; Sharma et al., 2022). In the present study, we address the research question of “What is the relation between patterns of community hospital procurement and prescription of antimicrobials, and how they shape AST-identified infection patterns?” Answering this question provides inputs to the development of antimicrobial stewardship policies and for building implications for policy and practice.

## 2 Materials and methods

### 2.1 Ethics declarations

The EquityAMR project is a 4-year collaborative study (2021–2025) between Norway and India focused on the analysis of health equities and AMR in India. The project obtained permissions from the Norwegian Centre for Research Data (NSD) and the Health Ministry Screening Committee (HMSC), Government of India, before data collection. For enabling data collection to ensure informed consent, anonymization, and adherence to required guidelines, formal ethical approval was obtained from the HISP India Research Ethics Committee. Memorandum of Understanding and Non-Disclosure Agreements were signed between HISP India and the state hospitals to ensure permissions for data collection and the security and integrity of data.

### 2.2 Study design and data collection

This study employed a cross-sectional observational design as part of the larger Equity AMR initiative, a collaboration between Norwegian and Indian researchers inspired by systems thinking to analyze the interconnected relationships between surveillance, diagnostics, and prescriptions. The study was conducted at a community hospital (CH), a secondary care public health facility in North India. Data were collected from three primary sources: (i) antimicrobial procurement records from the community hospital between 2018 and 2022; (ii) antimicrobial susceptibility testing (AST) results of clinical isolates from urine, pus exudate, and stool samples collected from outpatients who underwent microbiology culture tests between January 2023 and December 2024; and (iii) antimicrobial prescription data (2023) extracted from patient prescription slips following their OPD encounters at the hospital. Each dataset was analyzed both individually and in combination to provide a holistic understanding of hospital-level drivers of AMR and to draw policy-relevant implications.

### 2.3 Data collection of antimicrobial procurement

We collected data regarding inflow patterns of antimicrobials in the hospital, including from procurement to distribution within the facility. The data captured included the name of the healthcare community hospital, ordering time, generic names, dosage forms, strength, quantity purchased, expense, delivery time, etc. Data was then systematically collected from the manual stock ledgers in the stores.

For analysis, we compared the antimicrobials procured in terms of their compliance with the government-mandated Essential Drug List. We converted the compiled consumption data into defined daily doses (DDD) per 1,000 inhabitants per day, representing WHO’s standard measure (Misra et al., 2023). Based on 2011 census figures, we estimated the resident population for 2024. DDD per 1,000 inhabitants per day was used as a standardized metric, though in this hospital setting with a wider and partly undefined catchment, the values should be interpreted as approximate rather than precise. The DDD was calculated using the following formula:


$$\frac{\mathrm{Total}\,\mathrm{consumption}\,\mathrm{in}\,\mathrm{DDDs}}{\mathrm{Covered}\,\mathrm{inhabitants}\times \mathrm{Days}\,\mathrm{in}\,\mathrm{the}\,\mathrm{period}\,\mathrm{of}\,\mathrm{data}\,\mathrm{collection}}\times 1000$$

### 2.4 Antimicrobial prescription data

For prescription data, randomly selected patients accessing antimicrobials from the hospital pharmacy after an OPD encounter were approached. A total of 1,151 prescription slips were captured. Simple random sampling was employed, selecting slips that included one or more antimicrobials or names of infections that would potentially require future antimicrobial treatment. Random sampling was carried out to ensure that the selected prescription slips accurately reflected the entire group of patients using antimicrobials, thus decreasing biased selection. This methodology provides impartial and broadly applicable results about prescribing antimicrobial trends. Approximately 100 patients visited the pharmacy daily, from which the researcher, a trained microbiologist, randomly selected ∼10 patients, and consent was taken to examine their slips and take an image of them using a camera phone while covering patient names. The data collected included demographics, prescription details (symptoms, diagnosis, drug names, dosage, duration), and legibility of prescriptions. Data was recorded in Excel and then transferred into a bespoke database for analysis and interpretation. All three datasets, procurement, prescriptions, and AST results, were obtained from the same community hospital, with records cross-verified against pharmacy and laboratory registers to ensure consistency and reliability.

### 2.5 Clinical Data

The microbiology laboratory received 1,547 urine samples, 248 pus exudate samples, and 144 stool samples from patients attending the outpatient department (OPD) in the hospital. The samples were sent in for culture tests during the research period. However, blood samples were not tested as there is no facility for blood culture in this facility.

### 2.6 Clinical sample processing and bacterial identification

All clinical samples were processed in the Microbiology laboratory using conventional culture techniques. The urine samples were inoculated on Cysteine Lactose Electrolyte Deficient (CLED) agar medium. Inoculated agar plates were incubated initially aerobically at 37 °C for 24 h and finally for 48 h, and the plates were examined for pure growth. A growth of ≥ 10^5^ colony-forming units/ml was considered significant. Cultures with more than two colonies were considered contaminants, and such samples were discarded. Pus samples were processed for Gram staining and culturing. The samples were aseptically inoculated on blood agar (with 5% sheep blood) and MacConkey agar plates, incubated aerobically at 35 °C–37 °C for 24–48 h (Modgil et al., 2020). Stool samples were collected in sterile containers. They were cultured for the presence of bacterial species by standard procedures (Sharma et al., 2022). Briefly, samples were inoculated onto MacConkey agar, ampicillin blood agar, xylose lysine deoxycholate agar (XLD agar), thiosulfate-citrate-bile salts-sucrose agar (TCBS agar), alkaline peptone water (APW), and selenite F broth and incubated at 37 °C for 18–24 h. Pathogens were identified based on colony morphology, Gram staining, and standard biochemical tests such as indole, citrate, urease, triple sugar iron agar, and oxidase tests (Modgil et al., 2021).

### 2.7 Antibiotic susceptibility testing (AST)

Antibiotic susceptibility testing was performed with the disk diffusion method for the following antimicrobials: Amoxicillin + clavulanic acid, doxycycline, azithromycin, ampicillin, ciprofloxacin, amikacin, imipenem, levofloxacin, gentamicin, cefepime, piperacillin-tazobactam, ertapenem, cotrimoxazole, cefoxitin, ceftriaxone, etc., according to Clinical & Laboratory Standards Institute (CLSI) guidelines (Modgil et al., 2025). Quality control was performed using standard reference strains, including *Escherichia. coli* ATCC 25922 and *Staphylococcus aureus* ATCC 25923, to validate the potency of antibiotic disks, in line with CLSI guidelines.

### 2.8 Statistical analysis

Correlation analysis was conducted to explore potential associations between three variables: (i) antimicrobial procurement volumes (in DDDs), (ii) prescription frequencies of antimicrobials (percentage of total prescriptions), and (iii) AST-derived sensitivity percentages for selected organisms. Scatter plots were generated to visualize whether higher procurement or prescription corresponded with variations in sensitivity. These analyses were exploratory in nature and aimed to identify indicative trends and mismatches between antimicrobial use and resistance, rather than to infer causality.

## 3 Results

The results are presented at two levels. First, we provide a summary analysis of each of these three streams of data. Following this, we examine their inter-relationships.

### 3.1 Phase 1: independent analysis of the three data streams

#### 3.1.1 Antimicrobial procurement data

**FIGURE 1:**
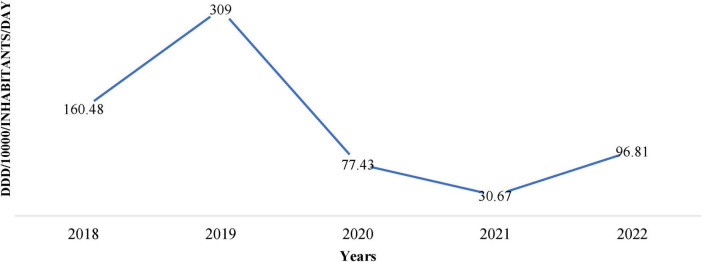
Institutional antimicrobial procurement in defined daily doses (DDD)/1,000/inhabitants/day over 5 years (2018–2022).

Figure 1 shows the trend of DDD per 1,000 inhabitants per day from 2018 to 2022 and depicts substantial variations with time. We observed a sharp increase from 160.48 DDD in 2018 to a peak of 309 DDD in 2019, followed by a decline to 77.43 DDD in 2020, reaching its lowest value of 30.67 DDD in 2021, as shown in Figure 1.

Table 1 represents data on the top Antimicrobials procured by the hospital during 2018–2022, including their class, AWaRe classification, route of administration, and procurement volume in DDD. Variations were observed, for instance, Azithromycin, classified as a Macrolide under the “Watch” category, showed varying procurement from 75717 DDD in 2018 to 22772 DDD in 2022. The dosage type of all the drugs was oral.

**TABLE 1:** Showing procured antimicrobials class, ATC classification, and AWaRe category.

Name of antimicrobial	Classof antimicrobial	ATC classification	AWaRe classification	Route of administration	Cost per antimicrobial dose to patients (INR)
Azithromycin	Macrolide	J01FA10	Watch	Oral	0
Doxycycline	Tetracycline	J01AA02	Access	Oral	0
Ofloxacin + ornidazole	Fluoroquinolone	J01MA01, P01AB03	Watch	Oral	0
Amoxicillin	Penicillin	J01CA04	Access	Oral	0
Amoxycillin + Clavulanic Acid	Penicillin + beta-lactamase inhibitor	J01CR02	Access	Oral	0
Cefixime	Cephalosporin	J01DD08	Watch	Oral	0

**FIGURE 2:**
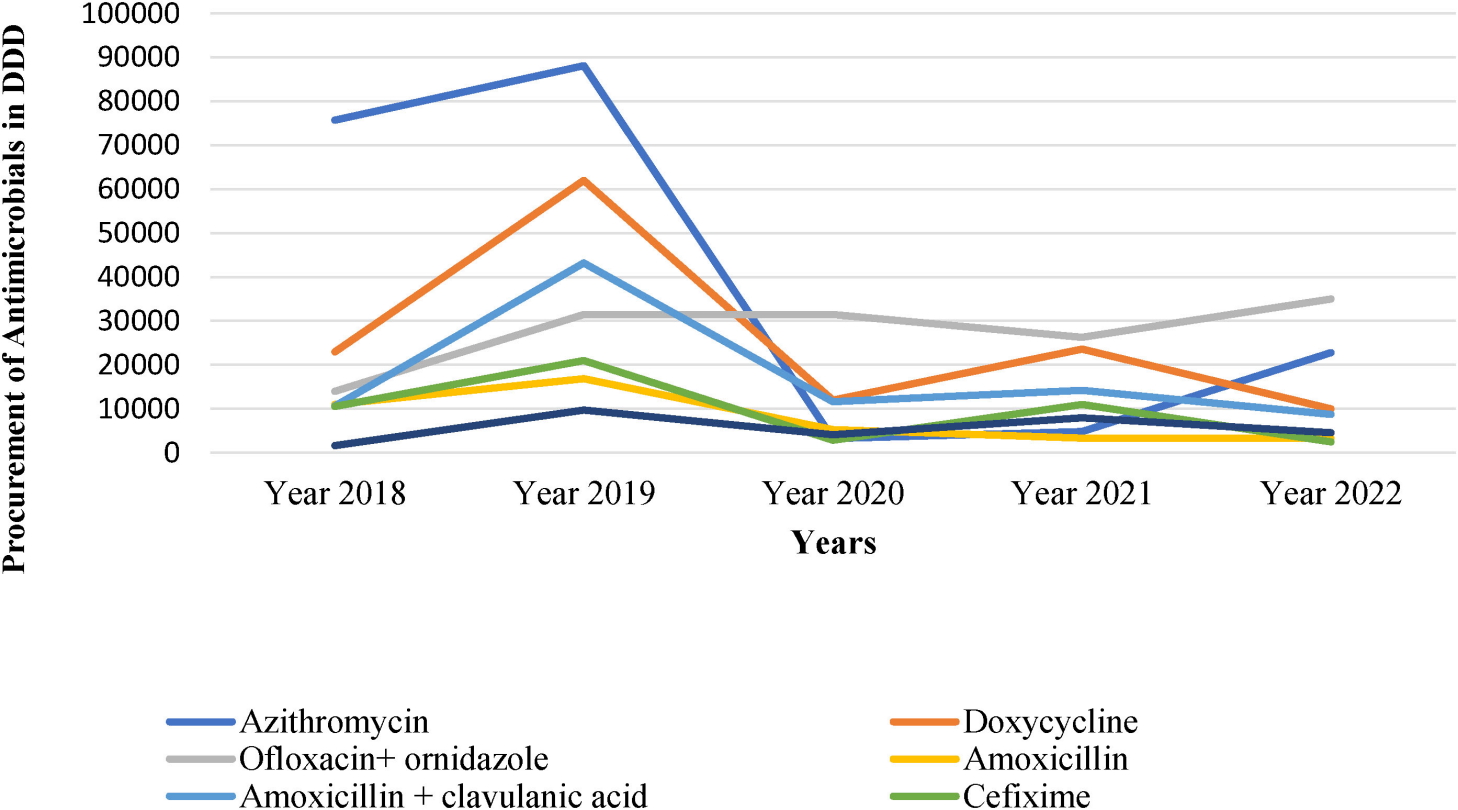
Top procured drugs over the year (2018–2022) in defined daily doses (DDD).

The antimicrobial procurement rate was the lowest in 2020, arguably attributed to COVID-19 when people were possibly avoiding hospitals and relying on self-medication. Similarly, other antimicrobials such as Doxycycline, Ofloxacin + ornidazole, Amoxicillin, Amoxycillin + Clavulanic Acid, Cefixime, and Metronidazole also showed fluctuations in procurement over the years (Figure 2).

These antimicrobials are procured by the district headquarters through a tendering process, based on their allocated budget. The drugs are then distributed to nearby secondary and primary health centers according to their needs and provided to patients free of charge (Table 1). In public health facilities in India, the cost of each prescription is frequently documented as “zero” as drugs are generally provided at no cost to patients through state health schemes. The initiative seeks to enhance healthcare accessibility and reduce the cost of treatment for citizens. This initiative is a component of the “Mukhyamantri Nirog Yojana,” which seeks to guarantee free accessibility to essential drugs and diagnostic services at government healthcare institutions. This program aligns with the underlying initiatives of the National Health Mission (NHM) intended to enhance public access to sustainable healthcare services.

### 3.2 Clinical data: AST patterns

**FIGURE 3:**
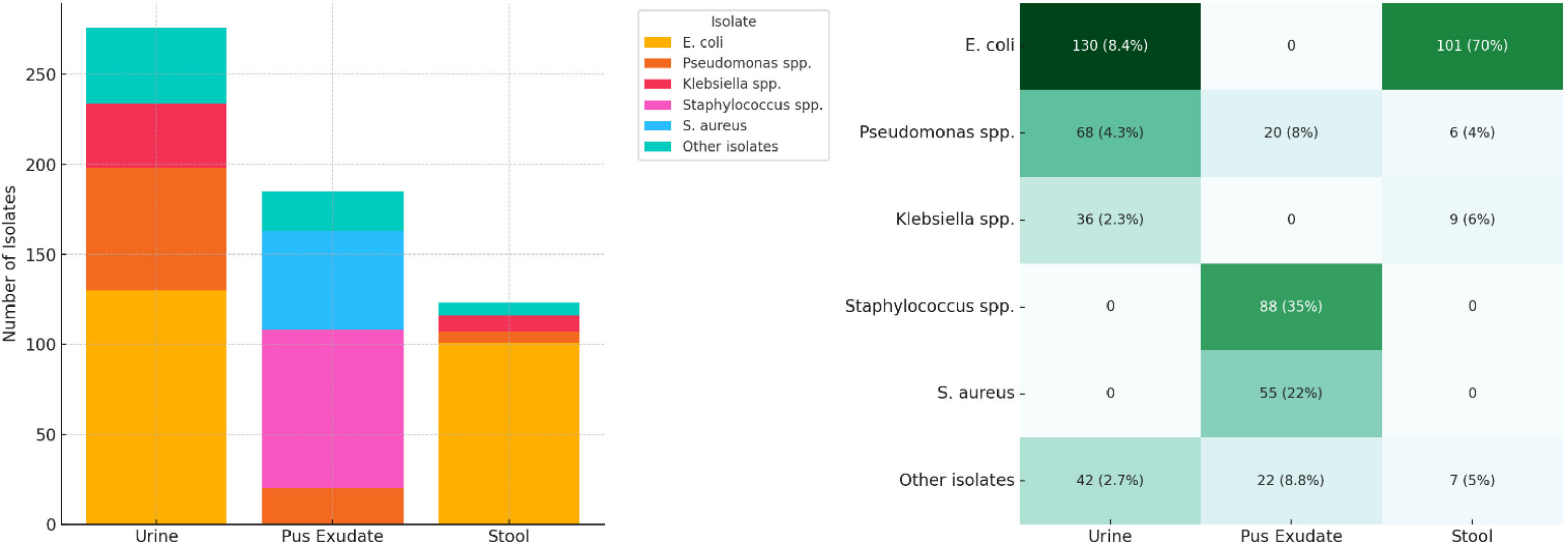
Clinical isolates from urine, exudate, and stool samples.

We compiled test results from a total of 1,547 urine samples, 248 pus exudate samples, and 144 stool samples processed in 2023. *E. coli* was the most prevalent organism isolated from urine (47.1%) and stool (69.7%) samples, while *Staphylococcus* spp. was the most isolated (46.8%) organism from pus exudate samples. The prevalence of *S. aureus* in pus exudate samples and the presence of *Salmonella* spp. in stool samples was 55 (29.3%) and 24 (16.6%), respectively. “Other isolates” referred to organisms detected in smaller numbers, such as *Proteus spp.*, *Enterobacter, Enterococcus spp.*, and *Candida spp.* The sample types tested, and their isolation rates are presented in Figure 3 below.

### 3.3 Antimicrobial prescription data

#### 3.3.1 Prescription and patient demographics

**FIGURE 4:**
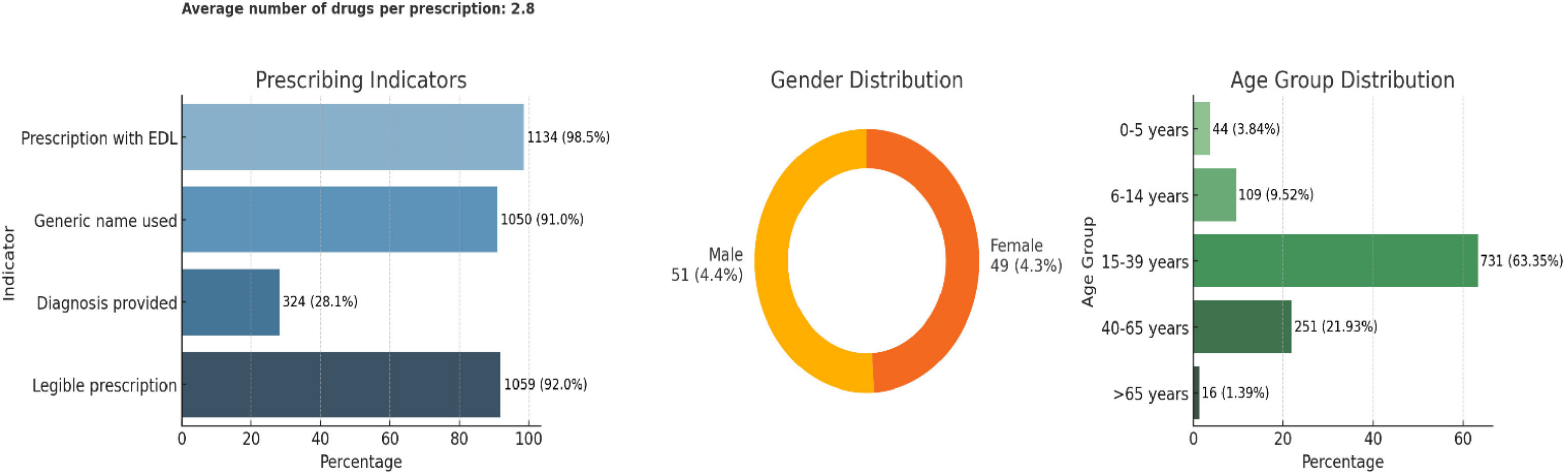
Key characteristics of antimicrobial prescriptions studied.

Among the 1,151 prescriptions analyzed, the average number of drugs per prescription was 2.8. Adherence to essential prescribing practices was high: 98.5% included drugs from the essential drug list (EDL), 91% used generic names, and 92% were legible. However, only 28.1% of prescriptions documented a diagnosis. In terms of patient demographics, 51% were male and 49% female. The majority of patients (63.4%) were in the 15–39 years age group, followed by 21.9% in the 40–65 years group, with minimal representation from the pediatric and elderly populations (Figure 4).

**FIGURE 5:**
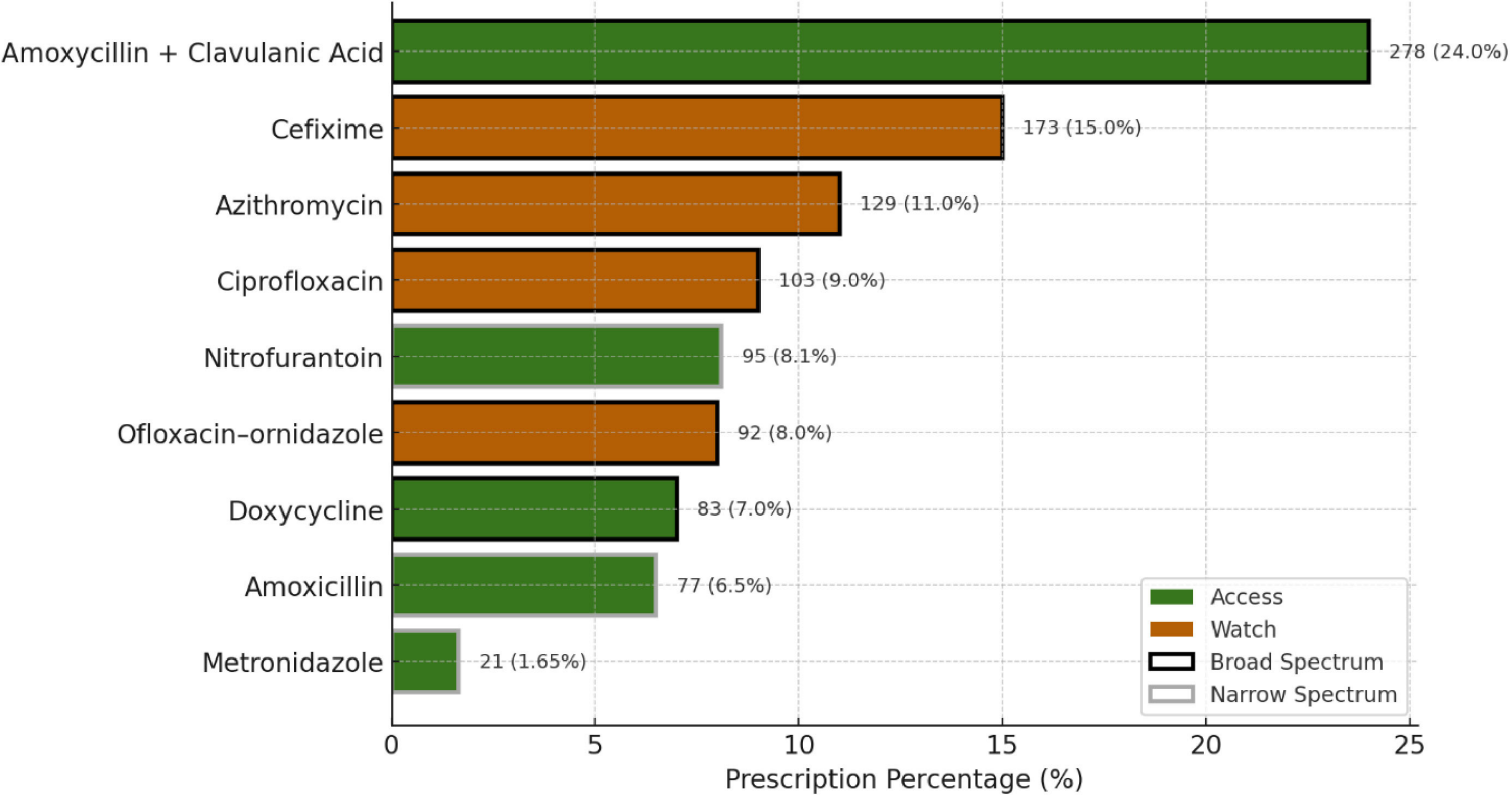
Patterns of antimicrobial prescriptions as per their class and WHO AWaRe classification.

The top three most prescribed antimicrobials were Amoxicillin + Clavulanic acid (24%), Cefixime (15%), and Azithromycin (11%), all of which are broad-spectrum antimicrobials and fall under the Access group of the AWaRe classification (Figure 5). All antimicrobials prescribed were from the Essential Drug List. Most prescriptions were from Dental and Dermatology outpatient departments, indicating a high usage of broad-spectrum antimicrobials. More than 50% of drugs were broad-spectrum, including Amoxicillin + Clavulanic acid, Cefixime, and Azithromycin.

### 3.4 Phase 2: integrated analysis

Table 2 presents the data between the top procured antimicrobials in the year 2022 in DDD/gm, the most prescribed antimicrobials in OPDs, and sensitivity results in 2023–24. Notably, Ofloxacin + ornidazole was the most procured antimicrobial, followed by Azithromycin and Doxycycline. Among the top prescribed antimicrobials in OPD in 2023, Amoxycillin + Clavulanic Acid had the highest prescription rate (24%), followed by Cefixime (15%) and Azithromycin (11%). Nitrofurantoin exhibited high sensitivity against *E. coli* (82.26%) and *Klebsiella* (65.7%) in urine samples, while Ciprofloxacin showed relatively low sensitivity (6.72% and 27.2%) against the same bacteria.

**TABLE 2:** Antimicrobial procurement, prescription, and sensitivity data.

Top 10 procured antimicrobials (year 2022)	Procurement of antimicrobials in DDD/gm	Prescription rate in OPD (2023), total patients *n* = 1,151	Antimicrobial sensitivity among clinical isolates (year 2023–24)			
			Antimicrobial sensitivity in *E. coli* (urine)	Antimicrobial sensitivity in *Staphylococcus* spp. (exudate samples)	Antimicrobial sensitivity in *Pseudomonas* spp. (urine)	Antimicrobial sensitivity in *Klebsiella* (urine)
Ofloxacin + ornidazole	35,000	92 (8%)	58.33% (49/84)	Not tested	Not tested	Not tested
Azithromycin	22,772	129 (11%)	32.43% (36/111)	38.21% (47/123)	Not tested	Not tested
Doxycycline	10,000	83 (7%)	61.74% (92/149)	93.22% (55/59)	Not tested	73.08% (19/26)
Amoxycillin + clavulanic acid	8,748	278 (24%)	47.06% (48/102)	75.86% (44/58)	Not tested	Not tested
Amoxicillin	3,350	77 (6.5%)	Not tested	66.67% (16/24)	Not tested	Not tested
Metronidazole	4,000	21 (1.65%)	Not tested	Not tested	Not tested	Not tested
Nitrofurantoin	3,500	95 (8.1%)	85.85% (182/212)	Only tested for urine isolates	76.67% (23/30)	72.09% (31/43)
Ciprofloxacin	3,125	103 (9%)	13.57% (27/199)	26.19% (33/126)	14.71% (10/68)	27.91% (12/43)
Cefixime	2,500	173 (15%)	26.83% (55/205)	Not tested	20.00% (6/30)	36.11% (13/36)
Norfloxacin	1,000	19 (1.44%)	45.81% (82/179)	Not tested	20.00% (6/30)	61.54% (24/39)
Ceftriaxone	730	3 (0.26%)	36.77% (57/155)	Not tested	Not tested	42.42% (14/33)
Cefuroxime	Not procured	12 (1.04%)	28.71% (58/202)	Not tested	Not tested	35.90% (14/39)
Cefixime -sulbactam	Not procured	3 (0.26%)	Not tested	Not tested	Not tested	Not tested
ofloxacin	Not procured	8 (0.7%)	58.33% (49/84)	Not tested	Not tested	Not tested

### 3.5 Statistical analysis

Correlation analysis was performed to assess associations between antimicrobial procurement volumes, prescription frequencies, and AST-derived sensitivity percentages. Scatter plots were used to visualize whether higher use corresponded with changes in susceptibility, highlighting mismatches such as the frequent prescribing of Amoxicillin–clavulanic acid alongside high resistance in *E. coli*. These findings are exploratory and should be interpreted as indicative trends rather than causal relationships, underscoring the need for richer longitudinal datasets. It is worth noting that, given the limited laboratory resources and incomplete testing across all antimicrobials and organisms, the available AST data were restricted. Therefore, there is a need for more tests and continuous data collection over longer time frames. As can be seen in Table 3, the most tests were conducted for *E. coli* in urine, across 9 (all except for Amoxicillin, Metronidazole, and Cefixime-sulbactam) of the 12 procured antimicrobials. *Pseudomonas spp*. in urine was tested across 4 (Nitrofurantoin, Ciprofloxacin, Cefixime, Norfloxacin), *Klebsiella* in urine was tested across 7 (Doxycycline, Amoxyclav, Amoxicillin, Metronidazole, Nitrofurantoin, Ciprofloxacin, Cefixime, Norfloxacin, Ceftriaxone, Cefuroxime), while Staphylococcus in exudate was tested in 5 (Azithromycin, Doxycycline, Amoxyclav, Amoxicillin, Ciprofloxacin). This results in data sparsity and limits the extent to which the data can be statistically analyzed.

**TABLE 3:** Correlation analysis of antimicrobial procurement, prescription, and sensitivity data, discarding clinical isolates and antimicrobials.

	2023–2024			2023			2024		
Variable	Procured	Prescribed	Sensitivity	Procured	Prescribed	Sensitivity	Procured	Prescribed	Sensitivity
Procured	1.0	0.2047	0.1522	1.0	−0.0233	0.2833	1.0	0.1497	0.0771
Prescribed	0.2047	1.0	0.0257	−0.0233	1.0	−0.2271	0.1497	1.0	0.0953
Sensitivity	0.1522	0.0257	1.0	0.2833	−0.2271	1.0	0.0771	0.0953	1.0

The primary analysis conducted was correlation analysis, over two conditions, to explore the nature of links between antimicrobial sensitivity and prescription:

1. Without specific diseases and antimicrobials – in this case, we discarded information on the type of antimicrobial sensitivity, and the antimicrobial and only focused on the relation between procurement, prescription and sensitivity metrics.
2. Including all details of diseases and antimicrobials – in this case, we preserved all the information on the sensitivity and the antimicrobial prescribed.

**FIGURE 6:**
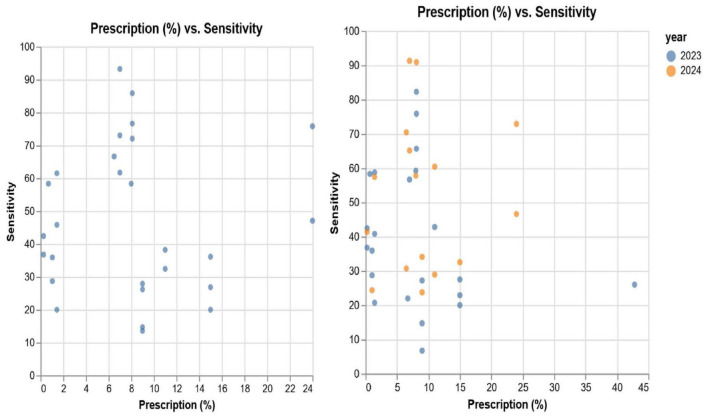
Scatterplot of prescription vs. sensitivity, comparing combined 2023/24 with 2023 and 2024.

Figure 6 presents a scatterplot of the prescribed antimicrobials (percentage) and sensitivity of all antimicrobials, with the 2023–24 data and split between 2023 and 2024.

The Table 3 presents the relationship between procured antimicrobials, prescriptions and sensitivity, discarding isolate and antimicrobial information. It is to be noted that the prescription data relates to 2023. As can be observed, we note low (positive) correlation between 2023 prescriptions and 2023–24 sensitivity. Despite this, we observe a weak negative correlation between 2023 prescriptions and 2023 sensitivity.

**FIGURE 7:**
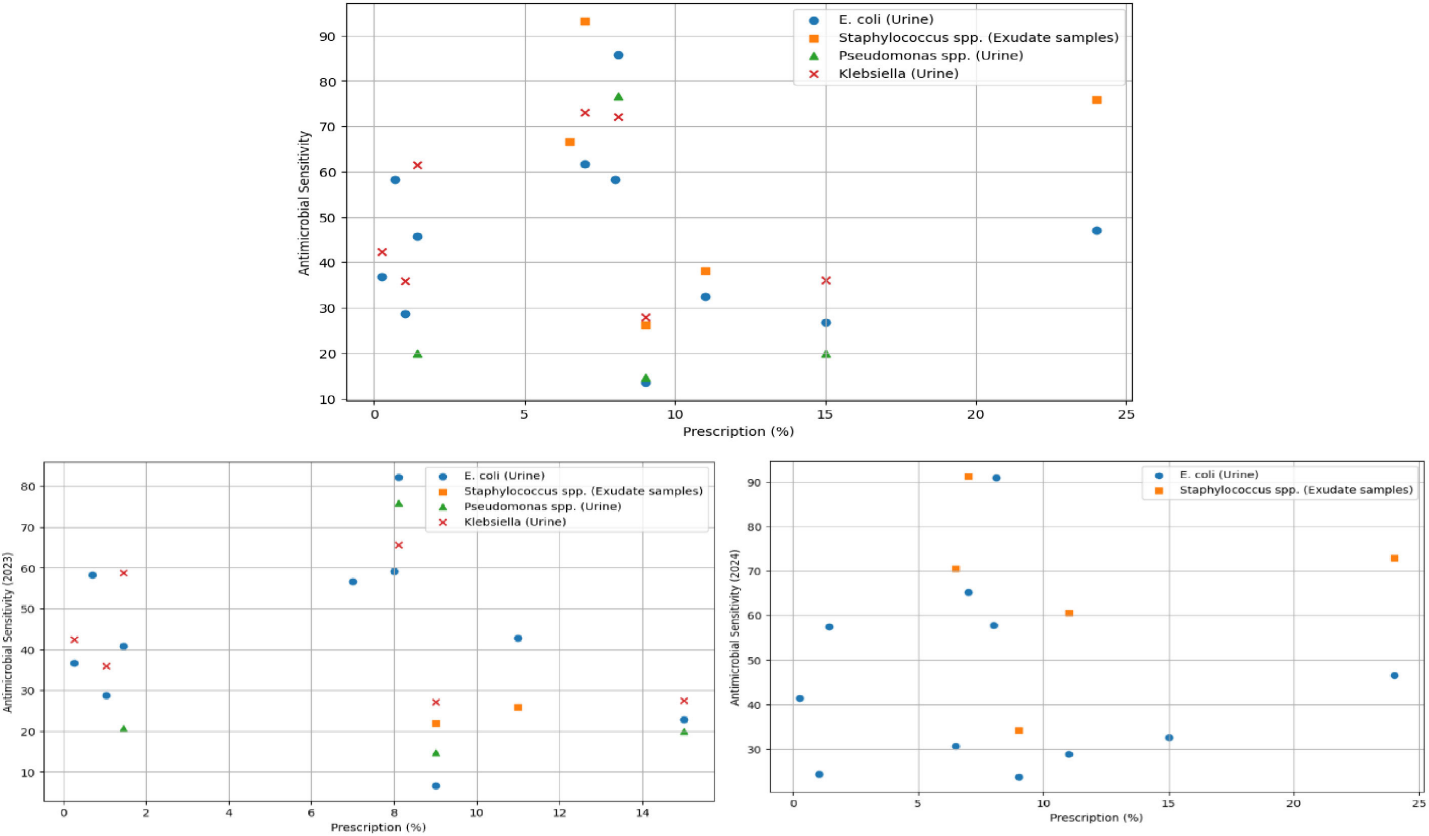
Scatterplot of prescription vs. sensitivity, grouped by microbe and clinical isolates.

To explore this further, we conduct correlation analysis between prescriptions and sensitivity, grouped by microbial sensitivities. We conduct this analysis on the sensitivity data for 2023–24 and, in Figure 7, we present a scatterplot of sensitivity vs. prescription, grouped by the microbe and isolates.

Table 4 presents correlation analysis of the antimicrobial sensitivity, procurement and prescriptions. The correlation values between pairs of microbial sensitivities are ignored in our interpretations as they do not correspond to the same populations, and therefore is not presented in the table.

**TABLE 4:** Correlation analysis of antimicrobial procurement, prescription, and sensitivity data, grouped into combined 2023/24, 2023, and 2024.

	Procured	Prescribed	*E. coli* (urine)			*Staphylococcus* spp. (Exudate samples)			*Pseudomonas* (urine)	*Klebsiella* (urine)
Variable			2023/24	23	24	2023/24	23	24	2023/24	2023/24
Procured	1.0	0.195	0.163	0.309	0.045	−0.125	1	0.164	0.534	0.504
Prescribed	0.195	1.0	−0.069	−0.130	−0.025	0.139	1	0.054	−0.040	−0.101

There are several key insights we derive out of the correlation analysis: inspecting the combined data, we observe a weak negative correlation (−0.069) between prescriptions and *E. coli* (urine) – as with the previous analysis, we note that the 2023 *E. coli* sensitivity (−0.13) is higher than 2024 (−0.025). In inspecting specific sensitivities, Staphylococcus spp. was determined to be weakly positively correlated (0.139) with prescriptions. The same was determined to be perfect correlation (1.0) with prescription for 2023. However, this is likely due to the sparsity of the data and only 2 of the 2 procured antimicrobials and therefore discard this as an observation. With more tests done in 2024, we observe a slightly positive correlation between *Staphylococcus spp*. and prescriptions. The number of tests for *Pseudomonas spp*. and *klebsiella* (urine) during 2024 were insufficient and combining 2023-24 data, both were determined as weakly negatively correlated with prescriptions, with −0.04 and −0.101 respectively. The negative correlations indicate that with an increase in the value of prescriptions, antimicrobial sensitivity reduces. However, given the limited and sparse nature of the data, longer term data collection is necessary to establish more generalizable findings.

## 4 Discussion

(i) Institutional procurement of drugs and AST result patterns

The present study depicts the link between the consumption of antimicrobials and the development of bacterial resistance. In our findings, Amoxycillin + Clavulanic acid, Ciprofloxacin, and Doxycycline were among the most procured drugs in the hospital, with a resistance rate of 53%, 87%, and 39% respectively, in *E. coli* (urine) isolates. Amikacin and Gentamicin were the least procured and had the lowest resistance, 5.5% and 10.3% in *E. coli* urine isolates, respectively. Also, high resistance rates were reported for moderately procured antimicrobials, Cefixime (74%) in *E. coli* isolated from urine samples. Prior studies have highlighted conflicting associations between antimicrobial procurement and resistance in bacterial isolates, both positive (Koneman et al., 2008) and non-existent (Baditoiu et al., 2017; CLSI, 2013; Colgan et al., 2008; Kahlmeter et al., 2003; Sun et al., 2012). Similar to our findings, Lee et al. (2013), Baur et al. (2017) have shown how hospital antimicrobial procurement practices have a major influence on the development of AMR in hospitalized patients (Hsu et al., 2010; Megraud et al., 2013). A study found that hospitals with procurement of broad-spectrum antimicrobials tend to have more prescriptions and higher rates of resistance, suggesting a link between procurement and resistance patterns (Lee et al., 2013). While an overall weak positive correlation was observed between antimicrobial procurement and sensitivity, this aggregate result masks important drug–pathogen–specific patterns that have greater clinical and stewardship significance. For instance, the high procurement and prescription of Amoxicillin–Clavulanic acid and Ciprofloxacin corresponded with markedly high resistance in *E. coli* isolates (53% and 87%, respectively). These specific negative associations provide more actionable evidence for stewardship than the global correlation, underscoring how frequent use of broad-spectrum and Watch-category antibiotics can reinforce resistance selection pressures. Therefore, while the integrated correlation framework demonstrates the feasibility of linking procurement, prescription, and resistance data, the interpretation has now been reframed to prioritize such clinically relevant mismatches over the overall correlation trend. The present study clarified that this correlation reflects an aggregated exploratory overview and is not intended to imply causation or clinical relevance. The drug- and organism-specific correlations can provide the more actionable and stewardship-relevant insights. This indicates that, whereas procurement influences resistance patterns, additional factors, including prescribing practices, misuse, and patient compliance, substantially impact AMR. These findings align with previous studies that have demonstrated a direct correlation between antibiotic consumption and the emergence of resistance. A study by Goossens (2009) found that countries with higher antibiotic consumption exhibited higher rates of resistance (Baur et al., 2017). Similarly, a study conducted in Korea observed a significant association between increased antibiotic usage and antimicrobial resistance among hospitalized patients (Al Masud et al., 2024).

This study also highlights the significant impact of the hospital antimicrobial formulary on prescription practices and resistance patterns. In this public system, the hospital formulary largely dictates prescribing, making procurement and prescription partially dependent. While this creates some circularity, analyzing them separately still helps identify mismatches between what is procured and what is prescribed, offering useful insights for stewardship. In our study, > 50% of the procured drugs belonged to a broad spectrum. Another important consideration is the costs of antimicrobial procurement, which also influences prescription patterns as hospitals often choose cheaper antimicrobials for procurement, which increases the prescription rates of these antimicrobials within the hospital. We reported that most medications procured by hospitals were from the EDL, and a large portion of prescriptions also aligned with the EDL list. This ensured that patients received medications at no cost. The zero-cost provision of medicines is also a key driver of consumption, as free access amplifies prescribing and uptake beyond the influence of formulary composition alone. The most procured and prescribed EDL antimicrobials were Azithromycin, Doxycycline, and Amoxicillin + Clavulanic acid. The cost of drugs, shaped by budget availability, significantly influenced procurement decisions, potentially compromising the quality and reliability of the drugs procured. Hospitals with a formulary that includes broad-spectrum antimicrobials are widely prescribed due to their availability and affordability, but their excessive use exerts selective pressure on bacteria, driving AMR. This is particularly concerning in settings where these drugs are provided at no cost, increasing their demand and overuse. Similarly, a study in Thailand found that hospitals keep the cost of antimicrobials low by widely available generics and government-produced drugs, making these medications affordable and accessible to most people. However, this widespread accessibility contributes to excess use and the development of AMR (Llor and Bjerrum, 2014).

(ii) Institutional procurement, prescription of drugs, and AST result patterns

Hospital antimicrobial prescription practices shape AST results, especially in severe infections where prompt treatment is crucial. In our study, the most procured drugs were azithromycin (11%), doxycycline (7%), and amoxycillin + clavulanic acid (24%), which were also the most prescribed by the physicians in the hospitals. amoxycillin + clavulanic acid resistance for *E. coli* (urine) was seen at 53%, whereas azithromycin resistance for *Staphylococcus* (exudate samples) was 62%. Our correlation analysis further demonstrated that *Staphylococcus spp.* in exudates was weakly positively correlated with prescriptions. Interestingly, the association in 2023 was weak, likely due to data sparsity, and hence cannot be considered an accurate reflection of current trends. However, with more tests conducted in 2024, a slight positive correlation was observed between *Staphylococcus spp.* sensitivity and prescriptions. This suggests that increased testing may provide more accurate insights into resistance patterns. These findings align with previous research indicating that prescribing patterns can significantly influence resistance trends. For instance, a study assessing antibiotic prescribing patterns using WHO prescribing indicators found deviations from recommended standards, emphasizing the need for improved antimicrobial stewardship programs to guide appropriate antibiotic use (Yin et al., 2018). In our findings, correlation analysis further revealed a weak negative correlation between prescription rates and *E. coli* sensitivity in urine samples. Similarly, *Pseudomonas spp.* and *Klebsiella spp.* in urine also demonstrated weak negative correlations with prescriptions, suggesting a gradual decline in sensitivity with increased antimicrobial use.

In addition, the WHO has set up a global target of greater than 60% use of access category antimicrobials. However, our hospital setting reported that > 90% of the procured drugs in the hospital (2018–2022) were from the Access category, much higher than the WHO target. No antimicrobials were procured from the reserve category. This highlights a limitation of the AWaRe metric, as broad-spectrum agents like Amoxicillin + Clavulanate fall under “Access”; hence, use must also be interpreted by spectrum and indication, not category alone. A study by Doi et al. (2017) showed the impact of incorporating AST data into empirical treatment algorithms, resulting in improved patient outcomes and reduced rates of antimicrobial resistance (Kim et al., 2018). Our prescription data were comparable to a study from Zawiya Teaching Hospital, Libya, where the most prescribed drug was Amoxycillin + Clavulanic Acid (31.3%) (Pokharel et al., 2024). Similarly, several studies have reported that increases in resistance rates were accompanied by prescriptions of non-restricted antimicrobials (Doi et al., 2017; Katakam et al., 2022). Numerous ecological studies have demonstrated that increased antimicrobial procurement is linked to the emergence of AMR in *Streptococci*, which is associated with the prescription of macrolides, especially azithromycin, a first-line medication prescribed for respiratory infections in the United States (Lim et al., 2014; van Buul et al., 2012). These comparisons suggest that resistance rates can vary significantly between different regions and populations, arising from different procurement and prescribing practices in hospitals (Goossens, 2009). Our findings emphasize the need for long-term surveillance, as the current dataset demonstrates fluctuating correlation trends between prescription rates and sensitivity across different years. The weak and inconsistent correlations observed suggest that additional factors, such as patient compliance, community-level antimicrobial use, and underlying resistance mechanisms, contribute to AMR. Strengthening laboratory infrastructure and increasing AST testing coverage will enhance the accuracy and utility of stewardship interventions. To demonstrate the practical value of this integrated approach, specific examples from the dataset were examined. Amoxicillin–Clavulanate showed high procurement (8748 DDD/gm) and frequent prescription (24%), yet only 47% of *E. coli* isolates were sensitive, indicating a clear mismatch between use and efficacy. Similarly, Ciprofloxacin demonstrated high usage with only 13.6% *E. coli* sensitivity, while Azithromycin (32.4%) and Cefixime (26.8%) also reflected poor sensitivity despite substantial procurement and prescription levels. These integrated findings highlight how linking procurement, prescription, and AST data can identify specific stewardship targets that remain obscured when analyzed independently. These findings suggest that while procurement and prescription patterns contribute to AMR, they are not the sole drivers, and additional factors such as infection control policies, community antibiotic use, and genetic mechanisms of resistance should be considered.

### 4.1 Implications for the development of antimicrobial stewardship programs (ASPs)

The development of ASPs in Indian institutions encounters several challenges, such as inadequate facilities, financial constraints, irregular prescription processes, and a lack of expertise and education among healthcare providers. The data gap is also a significant barrier because ASPs rely on accurate and up-to-date information to identify trends in AMR, monitor prescription practices, and optimize antibiotic use. While observational, our analysis directly points to actionable stewardship steps, aligning procurement with resistance, updating formularies, and integrating AST data into prescribing practice. A study carried out at a tertiary care hospital in India evaluated the impact of specific ASP strategies and infection control practices in ICUs on decreasing overall antimicrobial usage and infection rates through targeted interventions (García-Rey et al., 2006). Our study demonstrates several implications for the development of ASP strategies. First, there is a need for regular review and adjustment of the hospital antimicrobial formulary to ensure it aligns with current resistance patterns and stewardship goals. Second, integrating procurement, prescription, and sensitivity testing data can enhance the effectiveness of ASPs by providing a more comprehensive understanding of antimicrobial use and resistance trends. Third, addressing the economic and cost-effectiveness aspects of antimicrobial selection by monitoring and modifying procurement decisions. By using resistance surveillance data in ASPs, hospitals can develop evidence-based prescribing guidelines and combat the spread of resistant organisms, ultimately enhancing patient safety. Furthermore, in settings with high background resistance, promoting a premature shift toward narrower-spectrum “Access” drugs may risk treatment failure. A feasible first step is to strengthen diagnostic and AST capacity to generate reliable local antibiograms guiding safe de-escalation. Integrating this data into formulary and procurement decisions, introducing phased de-escalation in select departments, and maintaining clinician feedback loops can gradually reduce dependence on broad-spectrum “Watch” drugs while minimizing clinical risk.

### 4.2 Contributions

The findings from the two phases were summarized under three main categories: (1) Hospital procurement and AST patterns, (2) Hospital prescription practices and AST data, and (3) Development of ASPs through integrated data analysis.

1. Overall, our findings suggest that reducing the procurement of antimicrobials demonstrating high levels of resistance in the hospital could mitigate further increases in bacterial resistance. Also, the antimicrobial formulary in the hospital possesses significant impacts on prescribing practices and resistance patterns, emphasizing how formulary selections shape resistance development and spread. Cost factors have an important influence on the purchase of antimicrobials, which leads hospitals to choose cheaper but probably less effective medicines. This highlights the links between hospital funds and antimicrobial resistance in shaping resistance patterns.
2. Our findings suggest that the availability of resistance data within hospital settings serves as a crucial determinant of prescribing behaviors, as healthcare professionals rely on such data to guide empirical therapy. Our study indicates the need for further research in other geographical regions to study the epidemiological relationship between antimicrobial procurement and the emergence and transmission of resistant bacterial strains.
3. The combination of these three data types to enhance ASP effectiveness has not been considered, which is emphasized in this study. Our study highlights a comprehensive approach that integrates multiple sets of information to inform antimicrobial administration, correlate hospital prescription drug formularies to resistance patterns, and evaluate economic variables influencing antimicrobial selection to better regulate procurement decisions. Integrating data from three sources provides novel insights that would not be attainable by analyzing each data source independently.

### 4.3 Conclusion

To the best of our knowledge, this study is among the first from India to integrate antimicrobial procurement, prescription, and resistance data within a single institutional setting. Although constrained by its single-center design, outpatient focus, data sparsity in AST, and temporal disconnect between procurement and prescription/resistance datasets, it nevertheless demonstrates the feasibility of leveraging routine institutional data to identify mismatches and inform stewardship strategies. The absence of bloodstream isolate data highlights the need for strengthened laboratory capacity to capture the full resistance burden. The findings underscore the importance of aligning procurement with local resistance patterns, regularly updating formularies, and incorporating AST evidence into empirical prescribing. Future work should adopt standardized AST panels and include less-used drugs to reduce surveillance bias. Overall, the study highlights the critical need for synchronized, data-driven antimicrobial stewardship Programmes in which procurement, prescribing practices, and surveillance are continuously integrated to mitigate the escalating threat of AMR.

## Data Availability

The raw data supporting the conclusions of this article will be made available by the authors, without undue reservation.
